# Direct oral anticoagulant failure in stroke/transient ischaemic attack: neurologic and pharmacokinetic considerations

**DOI:** 10.1093/ehjcr/ytaa178

**Published:** 2020-08-21

**Authors:** David Z Rose, W Scott Burgin

**Affiliations:** Department of Neurology, Morsani College of Medicine, University of South Florida, 2 Tampa General Circle, Tampa, FL 33606, USA

**This editorial refers to ‘A case report of recurrent transient ischaemic attacks on dabigatran for atrial fibrillation: real-world insight into treatment failure’, by R. Huynh *et al.,* doi:10.1093/ehjcr/ytaa041.**

We read with interest the March 2020 article in *EHJCR* entitled, ‘A case report of recurrent transient ischaemic attacks on dabigatran for atrial fibrillation: real-world insight into treatment failure’.^1^ Huynh *et al.* report a case of ‘failure’ of a direct oral anticoagulant (DOAC) in an obese patient with presumed transient ischaemic attacks (TIAs). They describe symptoms of ‘intermittent dysgraphia and short-lived episodes of receptive dysphasia the prior week’ followed by awakening with ‘diffuse headache’ at 6:30 a.m. (on day of admission) and a ‘right superior visual field disturbance with colourful pixilation, lasting 2 min’ at 10.30 a.m., which recurred at 4.30 p.m. Authors labelled this panoply of symptoms as ‘recurrent TIAs’ and then based the patient’s DOAC compliance on a pharmacy-packed home medication box, as well as directly observed DOAC administration while hospitalized. After four supervised doses of the DOAC dabigatran 150 mg b.i.d., an assay revealed results below the minimum detection threshold (<40 ng/mL, Hemoclot, Hyphen Biomed).

As Vascular (stroke) Neurologists, we have concerns with: a) the depictions of TIAs and b) the assumption of a true failure of the DOAC . First, acute cerebral ischaemia has classic features of sudden functional loss: hemibody weakness/immobility, numbness/anaesthesia, speech/linguistic deficit, and/or visual hemi-field cut—termed ‘negative’ phenomena. Typically, such symptoms localize to a precise vascular territory in the brain or spinal cord. This patient’s symptoms, however, are varied, occur and recur over minutes, hours, or weeks, do not localize to one anatomical brain area, and include ‘positive’ phenomena (headache and pixilation) as well as stereotyped (occurring repeatedly in the same exact way) dysgraphia and dysphasia. Such cortex-based stereotypy can occur with focal, large-vessel, brain arterial pathology however this patient's reportedly ‘normal’ computed tomography angiogram (CTA) essentially excludes atherosclerotic or vasculitic intra- or extra-cranial stenosis.

Recurrent embolic episodes can provoke multifocal symptomatology, but again, do not explain the stereotypy or positive phenomena, and while not absolute, multiple recurrent events often result in some degree of magnetic resonance imaging (MRI) brain changes. We should expect at least one lesion to appear somewhere at some point on any MRI sequence (DWI, ADC, or FLAIR); however, this patient’s scan ‘did not show evidence of acute or recent infarct’. Combining all these elements together points us as Neurologists away from a diagnosis of stroke or TIA and much more towards migraine or partial seizure. The hallmark features of complex/complicated migraines (either new or chronic) are pixelated visual aura (scintillating scotomas, teichopsias, or photopsias to name a few) lasting minutes to hours to days, and often with a parade of other symptoms such as dysgraphia, dysphasia, and of course, headache. Conversely, TIA is seldom associated with acute cephalgia, and should not be favoured over an alternative headache diagnosis.^2^ Structural abnormalities (intracerebral haemorrhage, tumour, or cerebral venous sinus thrombosis) produce headache, but with an onset that may be insidious or prolonged, and were not evidenced here.

It is unclear if neurological expertise was involved in this case, as author affiliations are from the disciplines of Cardiology and Haematology. To quote a TIA review from experts in London, England: ‘There is no test for TIA: the gold standard remains assessment as soon as possible by a clinical expert… Interobserver agreement for the diagnosis of TIA between ' neurologists and non-neurologists is poor.^3^ Indeed, most patients presenting to specialty TIA clinics ultimately do not have a final diagnosis of TIA, with migraines being the most frequent mimic.^3^ To assist with TIA risk-assessment, neurologists include the standard, well-validated ABCD2 score (Age, Blood pressure, Clinical features, Duration, and Diabetes), which is not mentioned in this report; given the data presented, his ABCD2 score appears to be low (risk of impending stroke ∼3.1% within 90 days). Naturally, clinical differentiation of cases such as these may not be straightforward, so neurologist consultation is warranted; likewise, as neurologists, we consult our electrophysiologist when a stroke patient’s arrhythmia looks peculiar.

The second point worth mentioning is the Hemoclot assay. While dabigatran levels were subtherapeutic, we implore readers to consider more likely explanations than an unconfirmed polymorphism. Specifically for dabigatran, global pharmaceutical regulatory agencies have warned against the off-label use of a ‘pharmacy-packed medication box’ because this DOAC requires special packaging with desiccant due to its susceptibility to moisture and its potential to undergo acid hydrolysis, which can be accelerated by increased temperature.^4^ Dabigatran capsules should not be removed from the original blister pack for repackaging into ‘dose administration aids, dosette boxes, tablet organizers, or weekly medication packs’ like this patient did.^4^ It was not stated whether his medication and packaging were evaluated for such changes, nor if the four subsequent doses were from hospital supplies or home-packaged medication. Also, test timing (not specified peak vs. trough), and the duration of therapy (steady-state may require 3 days), may have impacted serologic levels.^5^

Nevertheless, this report is worthwhile: first, DOAC prescribers are reminded to use caution for patients >120 kg or body mass index >40 kg/m^2^ (he was 102.4 kg and 31.6 kg/m^2^). Second, DOAC ‘True failure’ does exist for both anti-FXa- and direct thrombin-inhibitors due to influence from other drugs and herbal remedies, especially those affecting the P-gp efflux pump system.^6^ Finally, as authors point out, there is a possibility of multiple pharmacogenomic factors that may impact drug metabolism.^7^ We agree that genetic analysis is sensible in certain situations of suspected DOAC ‘True failure’ in stroke associated with atrial fibrillation (AF)—but only after a comprehensive neurological review confirming the correct stroke/TIA diagnosis and excluding other non-AF aetiologies, plus assessment for alternative pharmacokinetic influences.

## Lead author biography

**Figure:**
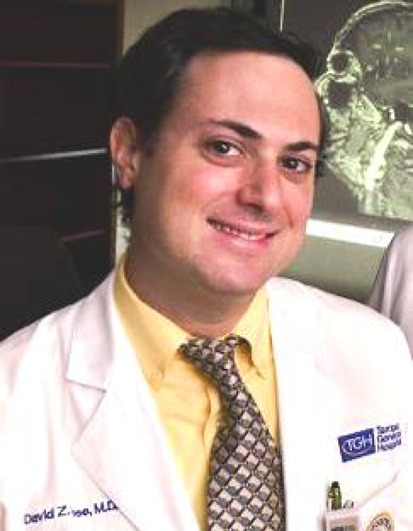


David Z. Rose, MD, trained in Stroke at University of Pennsylvania in Philadelphia, Neurology at University of Miami/Jackson Memorial Hospital, and Internal Medicine at Cleveland Clinic in Ohio. He is co-author of the fun review book "Laughing Your Way to Passing the Neurology Boards." Research interest of Dr Rose includes early DOAC use for Ischemic Stroke with Atrial Fibrillation (AREST trial), Hypertension management for Intracerebral Hemorrhage, and Synthetic Cannabis “Spice”-associated strokes.

**Figure:**
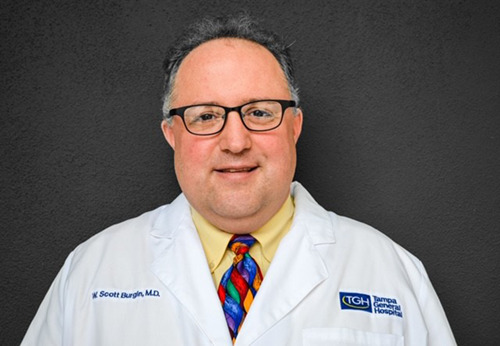


Dr W. Scott Burgin, MD, trained in Stroke at University of Texas Health Science Center in Houston and Neurology/Internal Medicine at University of Rochester in New York. Research interest of Dr Burgin includes early DOAC use for Ischemic Stroke with Atrial Fibrillation (AREST trial), Hypertension management for Intracerebral Hemorrhage, and Synthetic Cannabis “Spice”-associated strokes.

**Conflict of interest:** W.S.B. has received research support from BMS/Pfizer and VuEssence, and is on the Advisory Boards for Regeneron and VuEssence. D.Z.R. has participated in clinical trials with and received honoraria for advisory board/speaker’s bureau from Boehringer-Ingelheim, Boston Scientific, Medtronic, Chiesi, and CSL-Behring.

## Notes

The opinions expressed in this article are not necessarily those of the Editors of the *European Heart Journal* or of the European Society of Cardiology.
